# Pan-Cancer Analysis of NOS3 Identifies Its Expression and Clinical Relevance in Gastric Cancer

**DOI:** 10.3389/fonc.2021.592761

**Published:** 2021-03-04

**Authors:** Dan Zou, Zhi Li, Fei Lv, Yi Yang, Chunjiao Yang, Jincheng Song, Yang Chen, Zi Jin, Jinpeng Zhou, Yang Jiang, Yanju Ma, Zhitao Jing, Yu Tang, Ye Zhang

**Affiliations:** ^1^The First Laboratory of Cancer Institute, The First Hospital of China Medical University, Shenyang, China; ^2^Department of Medical Oncology, The First Hospital of China Medical University, Shenyang, China; ^3^Laboratory Animal Center, China Medical University, Shenyang, China; ^4^Lymphoma and Myeloma Diagnosis and Treatment Center, The Second Affiliated Hospital of Dalian Medical University, Dalian, China; ^5^Department of Respiratory and Infectious Disease of Geriatrics, The First Hospital of China Medical University, Shenyang, China; ^6^The First Department of Oncology, Shenyang Fifth People's Hospital, Shenyang, China; ^7^Department of Neurosurgery, The First Hospital of China Medical University, Shenyang, China; ^8^Department of Neurosurgery, Shanghai General Hospital, Shanghai Jiao Tong University School of Medicine, Shanghai, China; ^9^Department of Medical Oncology, Cancer Hospital of China Medical University, Liaoning Cancer Hospital & Insititute, Shenyang, China

**Keywords:** NOS3, pan-cancer, gene expression, gastric cancer, eNOS

## Abstract

**Background:** *NOS3* (endothelial NOS, *eNOS*) is a member of the nitric oxide synthase (NOS) enzyme family, mainly participating in nitric oxide (NO) generation. *NOS3* has been reported to inhibit apoptosis and promote angiogenesis, proliferation, and invasiveness. However, the expression pattern of *NOS3* and its diagnostic and prognostic potential has not been investigated in a pan-cancer perspective.

**Methods:** Data from the Genotype-Tissue Expression (GTEx), the Cancer Genome Atlas (TCGA), the Cancer Cell Line Encyclopedia (CCLE), and the Cancer Therapeutics Response Portal (CTRP) were employed and *NOS3* expression was comprehensively analyzed in normal tissues, cancer tissues, and cell lines. Immunohistochemical staining of tissue sections were used to validate the prognostic role of *NOS3* in gastric cancer patients. GSVA and GSEA analyses were performed to investigate signaling pathways related to *NOS3* expression.

**Results:** In normal tissues, *NOS3* was expressed highest in the spleen and lowest in the blood. *NOS3* expression was increased in stomach adenocarcinoma (STAD) and significantly associated with poor prognosis of patients. Immunohistochemical staining validated that *NOS3* was an independent prognostic factor of gastric cancer. Several canonical cancer-related pathways were found to be correlated with *NOS3* expression in STAD. The expression of NOS3 was related to the response to QS-11 and brivinib in STAD.

**Conclusions:** *NOS3* was an independent prognostic factor for patients with STAD. Increased expression of *NOS3* influenced occurrence and development of STAD through several canonical cancer-related pathways. Drug response analysis reported drugs to suppress NOS3. NOS3 might be a novel target for gastric cancer treatment.

## Introduction

*NOS3* (endothelial NOS, eNOS) is a member of the nitric oxide synthase (NOS) enzyme family, which is a cluster of catalytic enzymes that mainly participate in nitric oxide (NO) generation (1). The *NOS3* protein is encoded by the *NOS3* gene, located on chromosome 7q36.1. Usually, *NOS3* protein is constitutively expressed in cells in an inactive state. Following the increase in calcium (Ca^2+^) concentration in cells, it can be activated by combining with the CaM protein. In addition, the direct combination of *NOS3* with caveolin-1 (CAV-1) and heat shock protein 90 (HSP90) and the phosphorylation (Ser-1177) of *NOS3* by PI3K/Akt signaling can modulate the activity of *NOS3* protein (2–4).

*NOS3* protein was initially found to participate in NO generation, mainly in endothelial cells, and is associated with cardiovascular diseases such as hypertension, atherosclerosis, and diabetes mellitus (5). In recent years, *NOS3* has been found to play various roles in malignant tumors, such as inhibiting apoptosis and promoting angiogenesis, proliferation, invasiveness, and immunosuppression (6–8). Circulating *NOS3* levels were inversely correlated with progression-free survival and overall survival (OS) of metastatic colorectal cancer patients (9). Another research in mesenchymal colorectal cancer patients reported that *NOS3* upregulation occurs after Apc loss, which was associated with poor prognosis (10). In breast cancer, the increasing expression of *NOS3* was reported to be a pro-angiogenic factor (11). It was found to promote angiogenesis via PI3K/Akt/mTOR pathway, and enhance the migration and invasion via NOS3-NO-sGC-cGMP signaling in breast cancer cells (12, 13). In pancreatic cancer, *NOS3* promoted tumor maintenance through the PI3K-Akt-NOS3-RAS (wild type) pathway (14). *NOS3* inhibition by the inhibitor N(G)-nitro-L-arginine methyl ester (L-NAME) could suppress pancreatic ductal adenocarcinoma cancer (PDAC) tumor growth (15). *NOS3* activation was reported to promote the antiandrogen-resistant growth through NO-mediated AR suppression in prostate cancer (PCa) (16). And *NOS3* was found to participate in promoting aggressive phenotype of PCa, resulting in poor prognosis in PCa patients (17). In addition, Trachootham et al. found that non-tumorigenic epithelial cells from oral and ovarian tissue could be induced to become invasive in stroma containing p53-deficient fibroblasts, through NOS3/RNS/ICAM1 signaling (18). NOS3 was also found to participate in oncogenic inflammation and immunosuppression of tumors through NOTCH1-PI3K/AKT-NOS3 axis (19). *NOS3* expression inhibition was involved in cervical cancer cell sensitivity to radiotherapy (20). Additionally, many studies have reported that *NOS3* gene polymorphisms are associated with risk for cancer progression, cancer susceptibility and drug response (21–23). However, research by Smeda et al. reported that *NOS3* activity and phosphorylation reduction was an early event in the lung metastasis of breast cancer, preceding the onset of the mesenchymal phenotype (EndMT) (24). *NOS3* participated in the enhancement of Taxol chemosensitivity with astragaloside IV treatment in breast cancer as a downstream target of caveolin-1 (25). These studies suggest that *NOS3* may perform multiple functions depending on different tumor types, and genetic background. Studies on *NOS3* expression in tumors are still scarce, and the functions of *NOS3* in tumor pathogenesis, especially in gastric cancer, are not comprehensively understood.

By applying data from the Genotype-Tissue Expression (GTEx; https://www.gtexportal.org/home/), the Cancer Genome Atlas (TCGA; https://portal.gdc.cancer.gov/) and the Cancer Cell Line Encyclopedia (CCLE; https://portals.broadinstitute.org/ccle/), the expression level of *NOS3* in 30 different normal human tissues and 33 different tumors types, as well as the corresponding normal tissues and 1,457 cancer cell lines was systematically analyzed. We investigated the relationship between *NOS3* expression and the clinical phenotypes of patients for all cancers and then separately for each cancer type. Subsequently, GSVA and GSEA analyses were performed to investigate signaling pathways related to *NOS3* expression. Subsequently, *NOS3* protein level was individually assessed in gastric cancer tissues. Ultimately, the correlation between *NOS3* expression level in 664 cancer cells and cell response to 544 drugs was analyzed to explore the potential of *NOS3* as a therapeutic target.

## Materials and Methods

### Download of TCGA and GTEx Datasets

TOIL GTEx and TCGA (primary tumor and normal tissues) gene expression RNA-seq data (IlluminaHiSeq: log2-normalized_count+ 1) and TCGA phenotype data, containing 9359 TCGA tumor tissues, 727 TCGA normal tissues and 7792 GTEx normal tissues, were obtained from UCSC Xena (https://xena.ucsc.edu/). TOIL reprocesses raw GTEx and TCGA RNA-seq data to correct for batch effects and to allow for the merging of samples across GTEx and TCGA datasets for pan-analyses (26).

### Analyses of *NOS3* Differential Expression in Tumor and Normal Tissues

To analyze the differential expression of *NOS3* between TCGA tumors and normal tissues, *t*-test was applied for tumor types with at least two normal tissues. The median gene expression level was employed to calculate the fold change. Then, the log2-fold change (cancer vs. normal) was employed as the x-axis and -log10 *p*-value was employed as the y-axis to produce a Volcano plot for each tumor type. The expression profiles *NOS3* mRNA within and between tumor types were graphed using GraphPad Prism (version 7) (San Diego, CA, USA).

### Analyses of *NOS3* Expression and Clinical Phenotypes

*NOS3* expression levels among different tumor stages (TNM stage) were assessed by *t*-test (for two groups) and ANOVA analyses (for three and more groups). To assess the relationship of *NOS3* expression to overall survival (diagnosis to death), the median of *NOS3* expression in each tumor was used as cutoff value to divide patients into two groups, and Cox proportional hazards models were employed. OS time was defined as the time from the day at diagnosis to the date of death (dead patients) or the date of the last follow-up. Cox proportional hazards model was used to generate hazard ratio (HR) and 95% confidence interval (CI) for each cancer types. Kaplan-Meier survival analysis and the log-rank test was applied to calculate *p*-value. A forest plot was constructed to visually display the hazard ratio (HR) and 95% CI for each tumor type. *p* < 0.05 was considered a significant correlation. The survival curve and forest plot were generated by GraphPad Prism.

### GSEA and GSVA Analyses

Gene Set Enrichment Analyses (GSEA) was performed by GSEA v4.1.0 (www.broadinstitute.org/gsea) to detect discrepantly enriched signaling pathways between *NOS3* higher and lower group. The gene set “c2.cp.kegg.v7.1.symbols.gmt” from MSigDB gene set was selected as reference gene set. Signaling pathways with normalized *p* < 0.05, normalized enrichment score (NES) >1.5 and false discovery rate (FDR) *q* < 0.25 was considered as statistically significant.

We utilized the R package “GSVA” to perform Gene Set Variation Analysis (GSVA) analyses of *NOS3* expression to find the pathways most associated with *NOS3* expression. *P* < 0.05 was regarded as statistically significant. The gene set “c2.cp.kegg.v7.1.symbols.gmt,” was selected as the reference gene set. Signaling pathways commonly enriched by GSEA and GSVA analysis were considered to be potential pathways related to *NOS3* expression.

### Cell Lines *NOS3* Expression

*NOS3* mRNA expression, promoter DNA methylation, and copy number data were downloaded from the Cancer Cell Line Encyclopedia (CCLE, https://portals.broadinstitute.org/ccle/), which contained RNA-seq data, DNA methylation data from the matching reduced representation bisulfite sequencing (RRBS) and copy number data of 1,457 human cancer cell lines. *NOS3* expression levels among different cell lines of different cancer types were investigated. Box plots and scatter plots were downloaded from the CCLE website. The relation of *NOS3* mRNA to promoter DNA methylation and copy number was evaluated by Spearman correlation analysis.

### Drug Responses

The drug response data were obtained from the Cancer Therapeutics Response Portal (CTRP, https://portals.broadinstitute.org/ctrp.v2.1/), which contained the responses of 664 cell lines to 482 drugs. Spearman correlation analyses was also performed to evaluate the association of *NOS3* expression with drug responses (area under the curve, AUC) first for all cell lines together and then individually in STAD. Then, correlation *r*-value was employed as the x-axis and -log10 *p*-value was employed as the y-axis to produce a Volcano plot. NOS3 mRNA was used as x-axis and AUC was used as y-axis to generate a scatter plot. The volcano and scatter plots were plotted using GraphPad Prism.

### Immunohistochemical Staining and Result Analysis of Patients

In total, 90 clinical samples from gastric cancer patients were collected from the First Affiliated Hospital of China Medical University (Shenyang, China) from January 2013 to December 2014. Demographic and clinical characteristics such as age at initial diagnosis, gender, initial diagnosis date, and tumor stage were also collected. All the patients provided informed consent. And this study was approved by the ethics committee of the First Affiliated Hospital of China Medical University.

Formalin-fixed tissues were embedded in paraffin and cut into 5-μm thick sections for H&E staining and immunohistochemical staining. The expression level of *NOS3* was detected by streptavidin-peroxidase method. Antigen of de-waxed sections were exposed to 3% H_2_O_2_ for 10 min at room temperature to quench the endogenous peroxidase activity. Then the tissues were blocked with goat serum for 30 min at room temperature. After incubation with *NOS3* primary antibody (Abcam, 1:100) overnight at 4°C, tissues were incubated with the secondary antibody (10 min) and biotin-labeled horseradish peroxidase (10 min). Next, 3,30-diaminobenzidine tetrahydrochloride (DAB) kit (Maixin, China) was used to visualize the antibody binding. Eventually, immunohistochemical staining was observed under an inverted phase contrast microscope.

Immunoreactivity was dependently evaluated using semi-quantitatively method by two investigators. Five representative regions were randomly selected from the 400-fold field of view of the microscope. The immunoreactive score was determined by the proportion of positive cells and the staining intensity. The proportion of positive cells was scored as follows: <9%, 0; 10–25%, 1; 26–50%, 2; 51–75%, 3; 76–100%, 4. The staining intensity was scored as follows: 0 for no staining, 1 for light yellow, 2 for yellow, and 3 for brown. The final immunoreactive score was the product of the two scores.

### Statistical Analyses

For all statistical analyses, a *p* < 0.05 was considered statistically significant. All statistical analyses and visualization were accomplished by using GraphPad Prism 7 and R software (R version 3.6.0).

## Results

### *NOS3* mRNA Expression in Various Normal Tissues and Tumors

**Figure 1:**
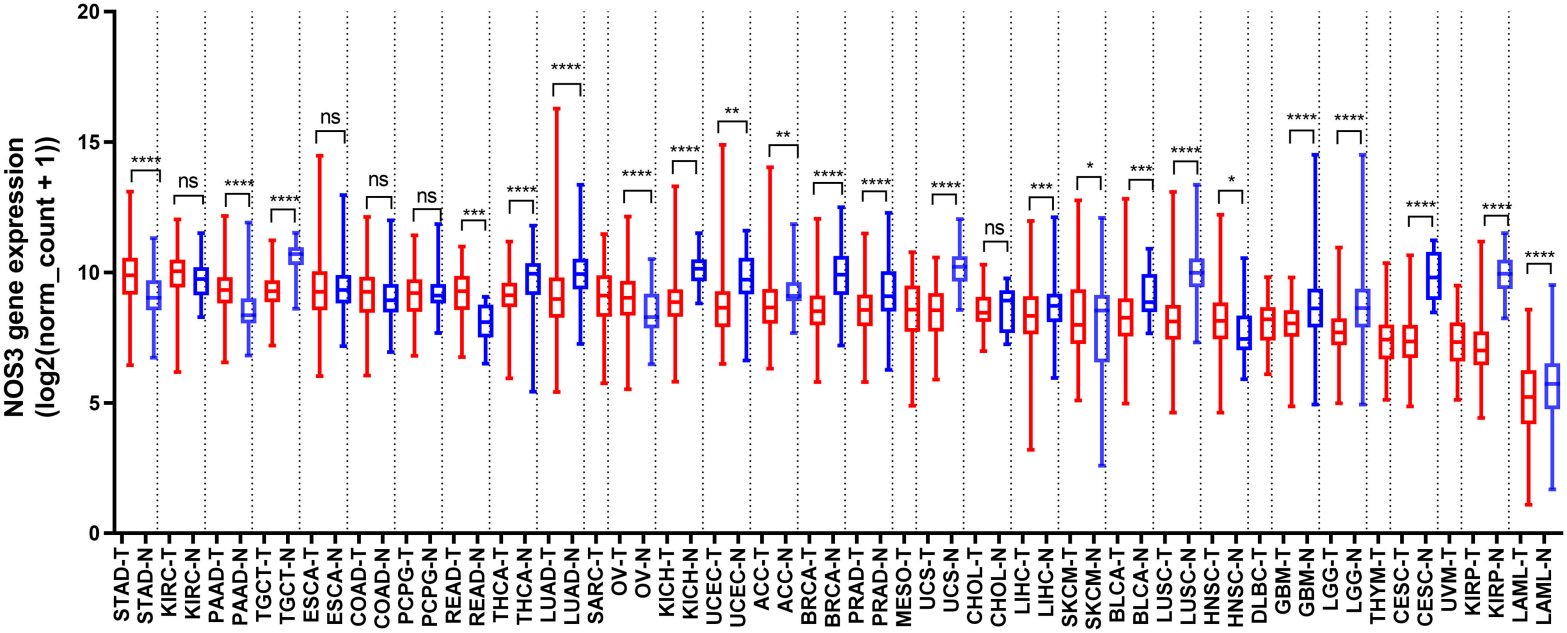
*NOS3* mRNA expression in various normal tissues and tumors. *NOS3* is differentially expressed between tumor and normal tissues in some cancers from TCGA and GTEx databases. Each boxplot represents *NOS3* expression [RNA-seq RSEM, log2(normalized count +1)] across different cancers. Red is for tumors and blue is for normal tissues. The bar represents median expression of tumors or normal tissues and lower and upper box ends represent the 25th and 75th percentile expression. ns, without statistical significance, *p < 0.05, **p < 0.01, ***p < 0.001, ****p < 0.0001, based on Student's *t*-test.

To comprehensively analyze *NOS3* expression and distribution in human normal tissues and tumor tissues, we first analyzed *NOS3* mRNA expression level in 30 different normal tissues from GTEx and 33 different tumor tissues from Xena (https://xenabrowser.net/). The expression of *NOS3* was highly variable across different normal tissues and tumor tissues (Figure 1, Supplementary Figure 1). In normal tissues, the median *NOS3* expression levels varied from 5.624 (blood) to 12.8 (spleen). Tissues with the highest *NOS3* expression were spleen (12.8 ± 1.391), heart (10.64 ± 1.099), testis (10.59 ± 0.532). Tissues with the lowest *NOS3* expression were blood (5.624 ± 1.325), skin (7.904 ± 1.753), pancreas (8.363 ± 1.159).

In tumor tissues, *NOS3* expression levels varied from 9.85 (stomach adenocarcinoma, STAD) to 5.071 (acute myeloid leukemia, LAML). Tumor tissues with the highest *NOS3* expression were STAD (9.85 ± 1.018), kidney renal clear cell carcinoma (KIRC, 9.848 ± 0.9791), pancreatic adenocarcinoma (PAAD, 9.297 ± 0.8167). Tumor tissues with the lowest *NOS3* expression were LAML (5.071 ± 1.591), kidney renal papillary cell carcinoma (KIRP, 7.173 ± 1.14), uveal melanoma (UVM, 7.278 ± 0.9764).

### *NOS3* mRNA Expression in Tumor Cell Lines

**Figure 2:**
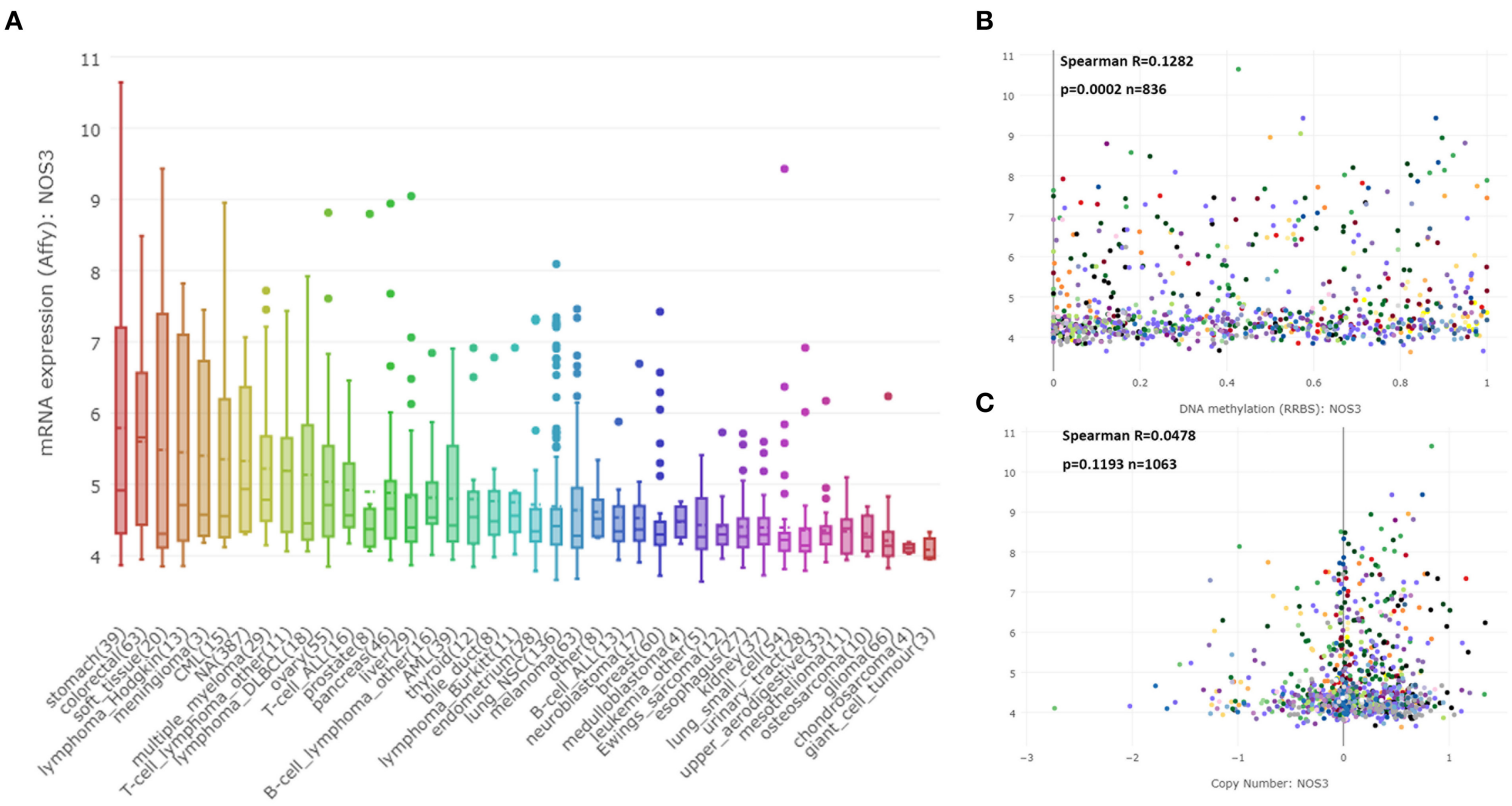
*NOS3* mRNA expression in tumor cell lines. **(A)** *NOS3* mRNA expression across different cell lines from CCLE. **(B)** A scatter plot of promoter DNA methylation and mRNA levels of *NOS3* across different cell lines is shown. **(C)** A scatter plot of copy number variation and mRNA level of *NOS3* across different cell lines. The correlation between two variables is analyzed by Spearman analysis.

Considering that tissue-based RNA expression detection might be complicated by the non-tumor tissues that are adjacent to tumor cells, we analyzed *NOS3* mRNA expression in 1457 cell lines derived from 26 tumor types in the CCLE database. Initially, *NOS3* expression in different cell lines was checked and the results showed that cell lines from STAD and COAD were the top two cell lines expressing the highest levels of *NOS3* mRNA, and cell lines from nerve system tissues (e.g., GBM and neuroblastoma) and bone tissues (e.g., chondrosarcoma and osteosarcoma) expressed relatively lower *NOS3* mRNA (Figure 2A). Interestingly, *NOS3* in STAD was expressed at the highest level both in stomach tissues from TCGA and in stomach cell lines from CCLE. Further analysis of the association between *NOS3* mRNA and promoter DNA methylation level showed a weak correlation (Spearman correlation coefficient = 0.1282, *p* = 0.0002, Figure 2B). Spearman correlation analysis between *NOS3* mRNA and copy number did not show statistical significance (*p* = 0.1193, Figure 2C). These results indicated that promoter DNA methylation and copy number variants of the *NOS3* gene might not be the main determinant of *NOS3* mRNA levels. *Tumor necrosis factor (TNF)-*α was reported to decrease functional activity of *NOS3* mRNA 3′-untranslated region (3′-UTR), regulating the translation process (27). In this research, we further determined the correlation between TNF-α mRNA and *NOS3* mRNA to verity if *TNF-*α affect transcription process of *NOS3*. However, there was only a weak correlation between them (spearman *r* = 0.1119, *p* = 0.003), suggesting that *TNF-*α might affect expression to a certain extent, but not a decisive factor (Supplementary Figure 2).

### *NOS3* Is Differentially Expressed in Various Tumors and Their Corresponding Normal Tissues

**Figure 3:**
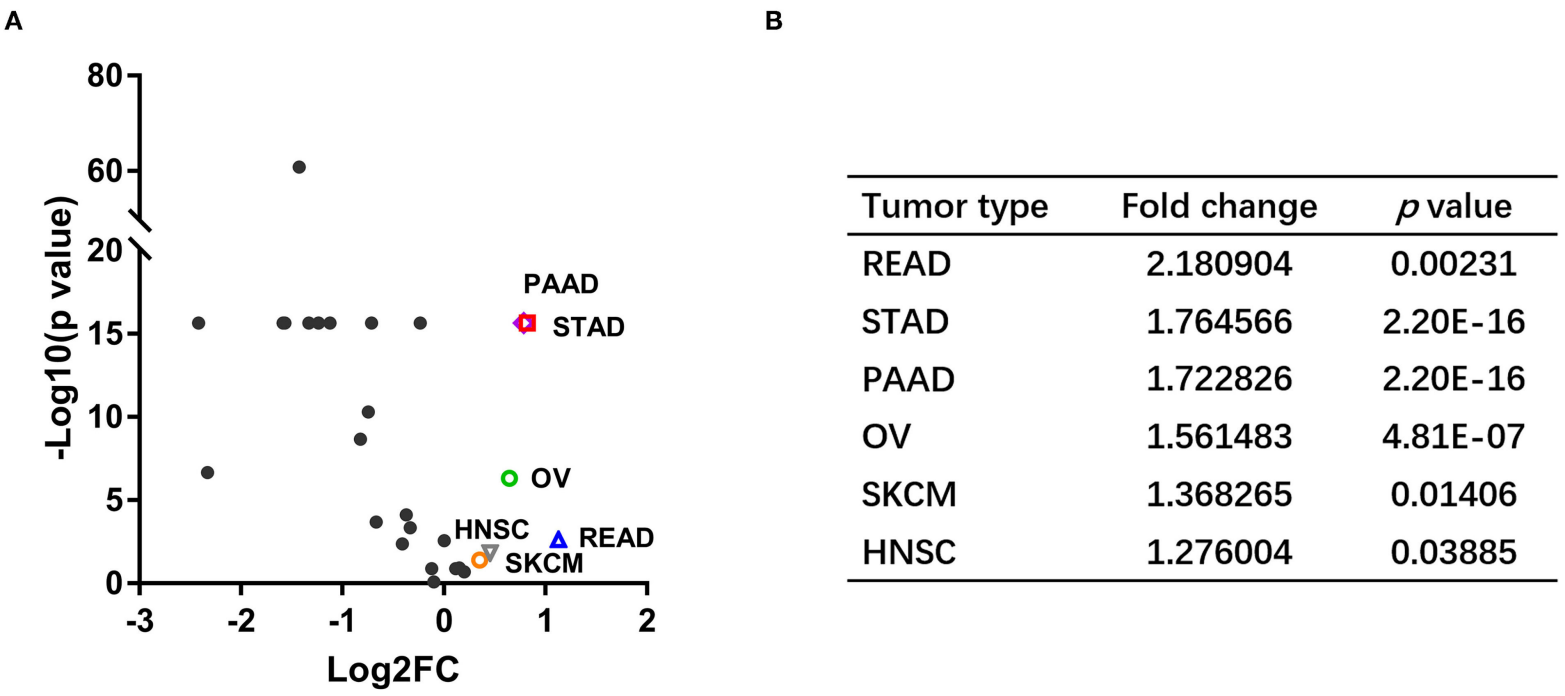
*NOS3* is differentially expressed in various tumors and their corresponding normal tissues. **(A)** Scatter plot of log2 FC and minus log10(*p*-value) across different cancers. The horizontal line on the Y-axis represents a *p-*value of 0.05. Points above the horizontal line have statistical significance. The vertical line on the X-axis represents log2 FC was−1 or 1, respectively. **(B)** The log2 FC and *p*-value in the six tumor types, which expressed higher NOS3 mRNA level.

We analyzed *NOS3* mRNA expression levels across tumors and their corresponding normal tissues in 28 tumor types that had three or more normal tissues data based on TCGA and GTEx database (Figure 1). *NOS3* mRNA expression in 6 of 28 tumor types, rectum adenocarcinoma (READ), STAD, PAAD, ovarian serous cystadenocarcinoma (OV), skin cutaneous melanoma (SKCM), head and neck squamous cell carcinoma (HNSC), was much higher than that in corresponding normal tissues, with statistical significance. Furthermore, we analyzed fold change (FC) of *NOS3* mRNA between tumor and corresponding normal tissues (Figures 3A,B). The FC in READ, STAD, PAAD, OV, SKCM, and HNSC ranged from 1.276 to 2.180. In cancer tissues and cancer cell lines, *NOS3* mRNA expressed highest in STAD. The differential analysis results showed that expression of *NOS3* in cancer tissues is 1.765-fold higher than that of corresponding normal tissues (*p* < 0.0001).

### Association Between *NOS3* mRNA Expression and Clinical Phenotypes

**Figure 4:**
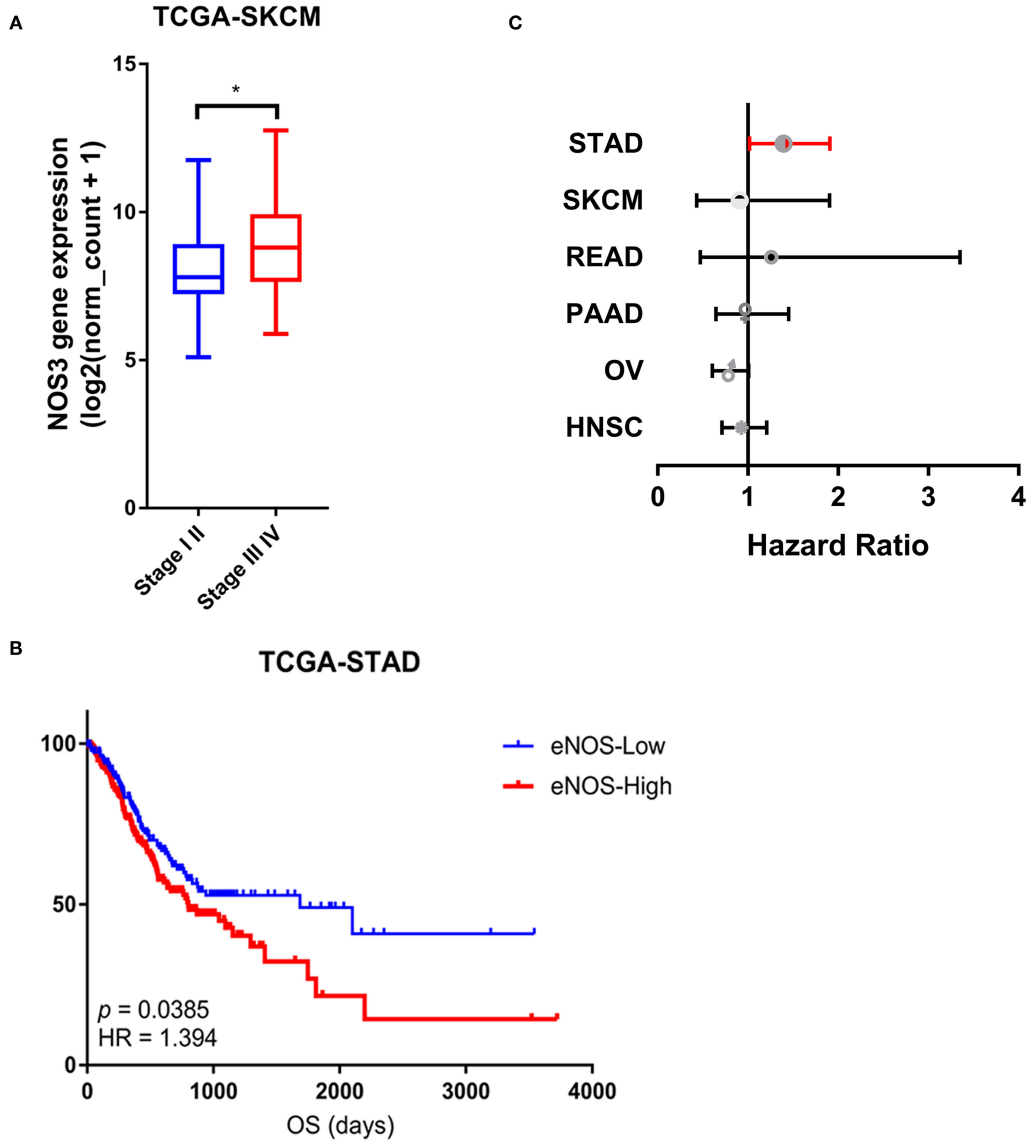
Association between *NOS3* mRNA expression and clinical phenotypes. **(A)** *NOS3* expression between the early and advanced stages in SKCM. *T*-test was applied to analyses in the early stage and advanced stage. *p < 0.05. **(B)** The *NOS3* expression level is related to overall survival in STAD. In the survival curves, the red line represents high *NOS3* expression levels and the blue line represents low *NOS3* expression levels. **(C)** A forest plot for survival association of each cancer is shown. The X-axis is the HR, the small points are the estimate of HR for each tumor and the bar represents the 95% confidence interval. Cox proportional hazards models were used to evaluate the association of *NOS3* expression levels on overall survival.

We analyzed the association between *NOS3* expression and tumor stage in 6 tumor types that had stage information in TCGA. Stages I and II were combined as early stage, and stages III and IV were combined as advanced stage. The result of *t*-test showed that in SKCM, patients with advanced tumor stage expressed higher *NOS3* mRNA levels, indicating that *NOS3* mRNA might positively related to later tumor stage (Figure 4A). However, *NOS3* expression in STAD patients did not show difference in early and advanced tumor stage (early stage: mean of *NOS3* expression = 9.888 ± 0.9934; advanced stage: mean = 9.872 ± 1.025, *p* = 0.6653) (Supplementary Figure 3). *NOS3* mRNA in READ, PAAD, OV, and HNSC also showed no correlation with tumor stage.

To analyze the relationship between *NOS3* expression and overall survival of tumor patients, log-rank test was performed in six tumor types. We found that among the six tumor types expressed higher *NOS3* level, *NOS3* mRNA was related to a worse prognosis in patients with STAD (median survival: 1,686 vs. 801, *p* = 0.0133385, HR = 1.394) (Figure 4B). However, *NOS3* mRNA did not show correlation with OS in READ, PAAD, OV, SKCM, HNSC (Figure 4C).

### Increased Expression Level of *NOS3* Was Related to Poor Prognoses of Gastric Cancer Patients

**Figure 5:**
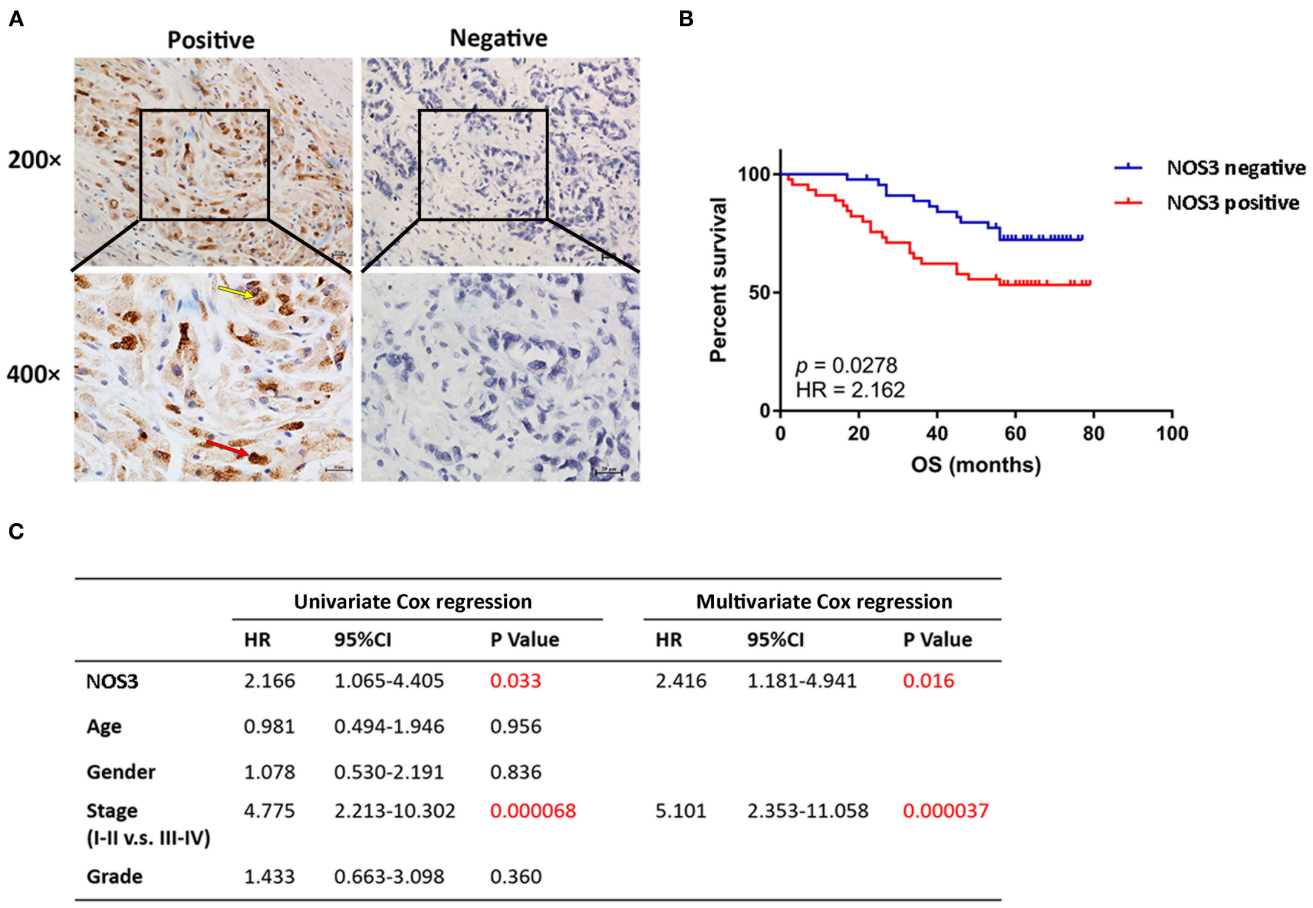
*NOS3* was independent prognostic factor of patients with gastric cancer. **(A)** Representative images of positive and negative *NOS3* protein expression in gastric cancer tissues. NOS3 was expressed in cytoplasm (yellow arrow) and nucleus (red arrow) of tumor cells. There were 45 patients stained positive and 45 patients stained negative. The scales bars indicate 20 μm. **(B)** Kaplan-Meier analysis of *NOS3* protein in gastric cancer patients. Patients with higher *NOS3* expression had shorter OS compared with patients with lower *NOS3* expression (*p* = 0.0278, HR = 2.162). **(C)** Univariate and multivariate cox regression showed that higher *NOS3* protein expression and advanced pathological stage were independent prognostic factor in gastric cancer patients.

Through the above pan-cancer analysis, we found that *NOS3* has a higher expression level in gastric cancer tissues, and it is significantly related to the poor prognosis of gastric cancer patients. Furthermore, the expression pattern of *NOS3* protein was explored in clinical tissue samples to validate the role of *NOS3* in gastric cancer. Based on *NOS3* protein expression levels, gastric cancer patients (*N* = 90) were divided into *NOS3* positive (*N* = 45) and *NOS3* negative (*N* = 45) group. The relationship of demographic and clinicopathological parameters with *NOS3* expression was analyzed using Chi square analysis. The results showed that *NOS3* expression was related to survival state (*p* = 0.049), but other parameters (gender, age, tumor stage, and grade) showed no correlation with *NOS3* expression (Table 1). Kaplan-Meier curves and log-rank test analyses confirmed that patients with positive *NOS3* expression had significantly shorter overall survival (OS) than patients with negative *NOS3* expression (*p* = 0.0278, Figures 5A,B). Furthermore, cox proportional-hazards model was used to validate the potential of *NOS3* as a prognostic factor in gastric cancer. Univariate cox regression suggested that *NOS3* expression (HR = 2.166, 95% CI: 1.065–4.405, *p* = 0.033) and tumor stage (HR = 4.775, 95% CI: 2.213–10.302, *p* < 0.001), were related to OS. Multivariate cox regression indicated that *NOS3* expression (HR = 2.416, 95% CI: 1.181–4.941, *p* = 0.016) and tumor stage (HR = 5.101, 95% CI: 2.353–11.058, *p* < 0.001) were independent prognostic factors for OS (Figure 5C). In summary, *NOS3* was an independent prognostic factor for patients with gastric cancer.

**Table 1:** Demographic and clinicopathological parameters of patients with gastric cancer.

		***N* = 90**	**NOS3 positive**	**NOS3 negative**	***p-*value**
Gender	Male	60	32	28	0.3711
	Female	30	13	17	
Age	<60	40	19	21	0.6714
	≥60	50	26	24	
T stage	T1-2	38	18	20	0.6695
	T3-4	52	27	25	
N stage	N0-1	62	27	35	0.0685
	N2-3	28	18	10	
pStage	I-II	50	23	27	0.3961
	III-IV	40	22	18	
Grade	1	6	3	3	1
	2-3	84	42	42	
Status	Alive	57	24	33	**0.049**
	Dead	33	21	12	

*The meaning of bold value is p < 0.05*.

### Mechanism of *NOS3* Influencing the Clinical Outcome in STAD

**Figure 6:**
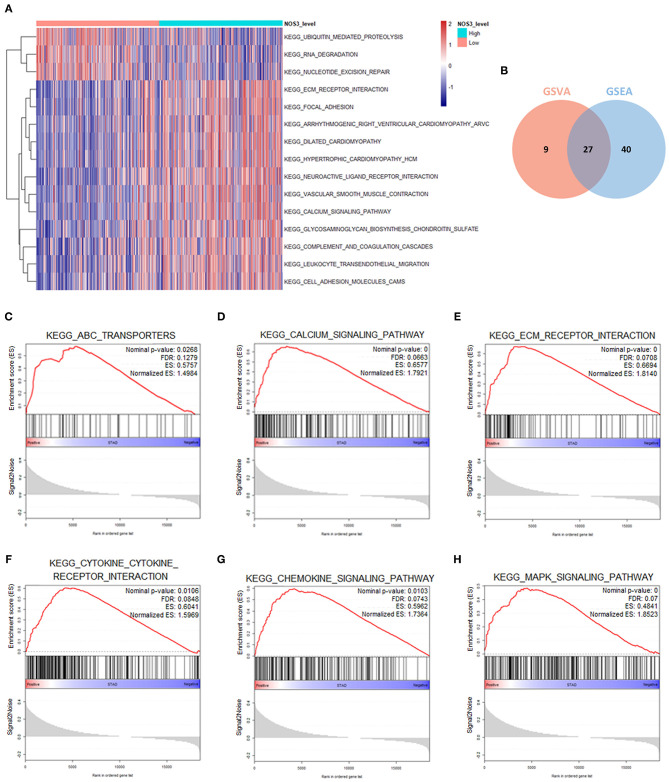
GSEA and GSVA analyses in STAD. **(A)** Top 15 differentially enriched KEGG signaling pathways in higher and lower *NOS3* expression groups analyzed by GSVA in STAD. **(B)** Seven signaling pathways commonly enriched by GSEA and GSVA in STAD. **(C–H)** Signaling pathways enriched by GSEA analyses in STAD.

In order to explore the mechanism underlying *NOS3* affecting clinical outcome of patients with STAD, GSEA and GSVA analyses were performed. Under the conditions of *p* < 0.05, FDR *q* < 0.25 and NES more than 1.5, GSVA and GSEA analyses commonly enriched 27 KEGG signaling pathways (Figures 6A–H, Table 2). Several canonical signaling pathways generally acknowledged to promote pathological behavior of malignant tumors were involved, such as “KEGG_ABC_TRANSPORTERS,” “KEGG_CALCIUM_SIGNALING_PATHWAY,” “KEGG_ECM_ RECEPTOR_INTERACTION,” “KEGG_CYTOKINE_ CYTOKINE_RECEPTOR_INTERACTION,” “KEGG_ CHEMOKINE_SIGNALING_PATHWAY” and “KEGG_MAPK_ SIGNALING_PATHWAY.” These results indicated that *NOS3* might participate in multiple canonical cancer-related signaling pathways to facilitate STAD.

**Table 2:** GSVA and GSEA analyses revealed mechanism of *NOS3* participant in occurrence and development of STAD.

**Signaling pathways**	**GSVA**	**GSEA**	
	***p*-value**	**NES**	***p*-value**
KEGG_GLYCOSAMINOGLYCAN_BIOSYNTHESIS_CHONDROITIN_SULFATE	<0.0001	1.48	0.044
KEGG_ECM_RECEPTOR_INTERACTION	<0.0001	1.81	<0.0001
KEGG_DILATED_CARDIOMYOPATHY	<0.0001	1.69	<0.0001
KEGG_VASCULAR_SMOOTH_MUSCLE_CONTRACTION	<0.0001	1.90	<0.0001
KEGG_NEUROACTIVE_LIGAND_RECEPTOR_INTERACTION	<0.0001	1.65	<0.0001
KEGG_CALCIUM_SIGNALING_PATHWAY	<0.0001	1.79	<0.0001
KEGG_FOCAL_ADHESION	<0.0001	2.01	<0.0001
KEGG_HYPERTROPHIC_CARDIOMYOPATHY_HCM	<0.0001	1.73	<0.0001
KEGG_CELL_ADHESION_MOLECULES_CAMS	<0.0001	1.71	0.006
KEGG_ARRHYTHMOGENIC_RIGHT_VENTRICULAR_CARDIOMYOPATHY_ARVC	<0.0001	1.63	0.004
KEGG_LEUKOCYTE_TRANSENDOTHELIAL_MIGRATION	<0.0001	1.99	0.002
KEGG_HEMATOPOIETIC_CELL_LINEAGE	<0.0001	1.60	0.033
KEGG_COMPLEMENT_AND_COAGULATION_CASCADES	<0.0001	1.58	0.012
KEGG_RENIN_ANGIOTENSIN_SYSTEM	<0.0001	1.54	0.025
KEGG_CYTOKINE_CYTOKINE_RECEPTOR_INTERACTION	<0.0001	1.60	0.011
KEGG_AXON_GUIDANCE	<0.0001	1.88	<0.0001
KEGG_CHEMOKINE_SIGNALING_PATHWAY	<0.0001	1.74	0.010
KEGG_PRION_DISEASES	<0.0001	1.51	0.037
KEGG_REGULATION_OF_ACTIN_CYTOSKELETON	<0.0001	1.87	<0.0001
KEGG_VIRAL_MYOCARDITIS	<0.0001	1.55	0.046
KEGG_DORSO_VENTRAL_AXIS_FORMATION	<0.0001	1.77	0.002
KEGG_MAPK_SIGNALING_PATHWAY	<0.0001	1.85	<0.0001
KEGG_ALDOSTERONE_REGULATED_SODIUM_REABSORPTION	<0.0001	1.50	0.035
KEGG_TYPE_II_DIABETES_MELLITUS	<0.0001	1.59	0.012
KEGG_GAP_JUNCTION	<0.0001	1.65	0.004
KEGG_ABC_TRANSPORTERS	<0.0001	1.50	0.027
KEGG_ADIPOCYTOKINE_SIGNALING_PATHWAY	<0.0001	1.62	0.019

### Association Between *NOS3* Expression and Drug Sensitivity

**Figure 7:**
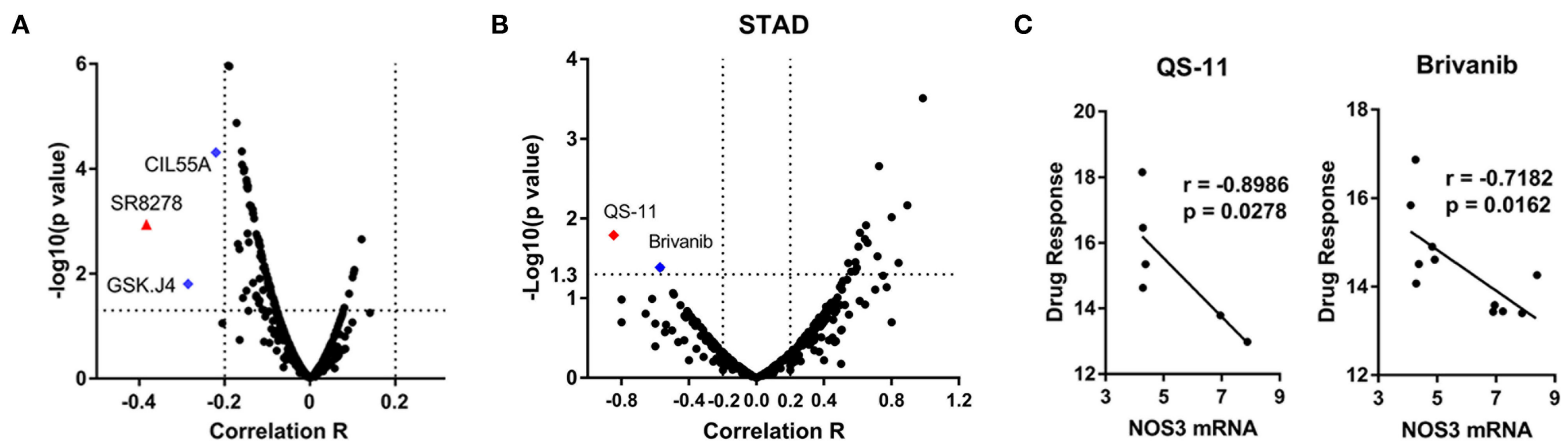
Association between *NOS3* expression and drug sensitivity. **(A)** Volcano plot of the correlation coefficient and minus log10(*p*-value) between *NOS3* expression in all cell lines and 482 drugs. Most of correlations are not significant and in negative direction. The correlation coefficient of “SR8278” is >0.3 (negative, higher expression is correlated with better response represented by smaller AUC). Blue dots are the drugs with correlation coefficients >0.2. **(B,C)** Spearman analysis performed individually in STAD. Volcano plot of the correlation coefficient and minus log10(*p*-value) between *NOS3* expression in STAD cell lines was shown in B (including 23132-87, NCI-N87, MKN-45, MKN-1, HS746T, NUGC-3, MKN-7, IM-95, HGC-27, OCUM-1, FU-97, and AGS cells). The response of cells to QS-11 (correlation coefficient *r* = −0.8986, *p* = 0.0278) and brivanib (correlation coefficient *r* = −0.7182, *p* = 0.0162) was significantly correlated with *NOS3* mRNA level. The scatter plots of QS-11 and brivanib were shown in **(C)**.

To investigate the correlation between *NOS3* mRNA expression and drug sensitivity, *NOS3* expression in 664 cell lines and drug response to 482 drugs were analyzed. Spearman correlation analysis revealed that, “SR8278” was considered to moderately correlate with *NOS3* mRNA expression, with a correlation coefficient >0.3 (Figure 7A). A negative correlation indicated that a better response (smaller response AUC value) was correlated with increased expression of *NOS3*. “SR8278” is an antagonist of the transcription factor REV-ERBα, affecting its circadian and metabolic functions. Two other drugs, “GSK.J4” and “CIL55A” had a correlation coefficient >0.2 and were also negatively correlated. Subsequently, Spearman analysis were performed to investigate the correlation of *NOS3* expression with drug response individually in STAD. The results showed that response of cells to QS-11 (correlation coefficient *r* = −0.8986, *p* = 0.0278) and brivanib (correlation coefficient *r* = −0.7182, *p* = 0.0162) was significantly correlated with *NOS3* mRNA level (Figures 7B,C).

## Discussion

*NOS3* has been found to inhibit apoptosis and promote angiogenesis, proliferation, invasiveness, and immunosuppression of malignant tumors. However, because of the limited number of studies on *NOS3* expression in malignant tumors, *NOS3* functions in tumor pathogenesis and development are still not fully understood. And the expression pattern of *NOS3* and its diagnostic and prognostic potential has not been investigated in a pan-cancer perspective. In this study, the expression level of *NOS3* (mainly mRNA) in 30 different normal human tissues, 33 different tumors types as well as their corresponding normal tissues, and 1,457 cancer cell lines was systematically analyzed, to determine the expression level of *NOS3* in tumor and normal tissues and its role in malignant tumors. We also explored its potential association with clinical characteristics (pathological stage, OS and drug response).

Pan-cancer analysis focused on whole genome can reveal genes that are associated with the occurrence and development of cancer, providing insights into cancer diagnosis, monitoring and treatment (28–30). By analyzing *NOS3* mRNA levels in normal tissues from GTEx, we found that *NOS3* was expressed at the highest level in the spleen and was expressed at the lowest level in the blood. According to the Human Protein Atlas (www.proteinatlas.org) database, the *NOS3* protein level in the spleen was also the highest, which was consisted with our results. And research has reported that *NOS3* is mainly upregulated in endothelial progenitor cells (EPCs) of the spleen, exerting beneficial functions on atherosclerosis, angiogenesis, and vascular repair (31, 32). And in 33 tumor types involved in this study, *NOS3* mRNA was expressed highest in STAD. Analyses in cancer cell lines showed that *NOS3* was expressed at quite high levels in COAD and STAD cell lines. Previous research showed that *NOS3* promoter DNA methylation could reduce *NOS3* mRNA level (33). Copy number variations (CNVs) could also modify gene mRNA expression (34). However, further analyses about promoter DNA methylation and CNVs of *NOS3* gene suggested that neither of the two factors showed a strong statistical correlation with *NOS3* mRNA, indicating that promoter DNA methylation and CNVs of the *NOS3* gene might not be the main determinant of *NOS3* mRNA levels in tumors.

Analyses of TCGA data showed that *NOS3* expression increased in six tumor tissues compared with their corresponding normal tissues. Among the six tumor types, NOS3 related to advanced tumor stage in SKCM. Previous research reported by Panich et al. suggested that NOS3 inhibition could effectively protect against UVA-dependent melanogenesis (35). In addition, patients with higher *NOS3* levels were diagnosed with a later tumor stage in COAD (Supplementary Figure 4A). This was consistent with the observation that L-NIO (a *NOS3* inhibitor) inhibited cell growth and angiogenesis in colorectal cancer (36, 37). In BRCA, we also found that the higher expression of *NOS3* mRNA was related to advanced tumor stage (Supplementary Figure 4B). These results were consistent with previous researches, which reported that *NOS3* promoted angiogenesis and enhance the migration and invasion in breast cancer cells (11–13).

There are few researches have reported *NOS3* in STAD. Doi et al. reported in 1999 that the quantity of *NOS3* in gastric cancer tissues was negatively correlated with serosal invasion (38). And *NOS3* has been reported to promote the angiogenic phenotype and predict poor prognosis in STAD (39). In our research, expression level of *NOS3* was significantly increased in STAD tumor tissues, and its expression level was the highest among the tumor types and cancer cell lines involved in this study. Analyses of clinical parameters also showed that *NOS3* predicted poor prognosis, consistent with previous research. These results confirmed the important role of *NOS3* in the development of STAD. Furthermore, experiments and analyses in our gastric cancer tissues also indicated that higher *NOS3* protein level was closely related to shorter OS of gastric cancer patients. *NOS3* was an independent prognostic factor for patients with gastric cancer. However, *NOS3* showed no correlation with tumor stage in mRNA and protein level. It may be due to the limitation of sample size. The sample size needs to be increased for further verification in future research. At present, the mechanism of *NOS3* promoting STAD progression was not clear. Therefore, we further analyzed the potential signaling pathways participating in *NOS3* promoting gastric cancer. The results of GSEA and GSVA analyses suggested that in STAD, several canonical cancer-related pathways were enriched in higher *NOS3* expression group. As key members of “KEGG_ABC_TRANSPORTERS,” ATP binding cassette (ABC) transporters were identified to mediate multidrug-resistance (MDR) in acute myeloid leukemia (AML), OV, BRCA, and lung cancer (40, 41). And researches also reported that ATP-binding cassette transporter G1 (ABCG1), a member of ABC transporter family, could modulate the interaction of Cav-1 and *NOS3* protein in endothelial cells (ECs), and increase cell migration through Lyn/Akt/*NOS3* in endothelial progenitor cells (EPCs). “KEGG_CALCIUM_SIGNALING_PATHWAY” contributes to many crucial tumor pathology processes, including proliferation, invasion, cell death, and autophagy in many tumors (42–45). As described above, the increased concentration of Ca^2+^ could induce the combination of CaM protein and *NOS3* protein, and subsequently stimulate the activity of *NOS3* protein (2, 46). We also enriched “KEGG_ECM_RECEPTOR_INTERACTION.” Specific interactions between the extracellular matrix (ECM) and cells are mediated by transmembrane molecules, including integrins, proteoglycans, and other cell-surface-associated components. These interactions can lead to malignant biological behavior of tumor cells, such as adhesion, migration, proliferation, and apoptosis (47, 48). A study by Njah et al. found that Agrin interacted with Lrp4-Integrin β1-FAK axis in ECs. This interaction could sustain the VEGFR2 pathway as well as stimulate *NOS3* signaling, and ultimately promote angiogenesis in tumor (49). In addition, “KEGG_CYTOKINE_CYTOKINE_RECEPTOR_INTERACTION,” “KEGG_CHEMOKINE_SIGNALING_PATHWAY,” and “KEGG_MAPK_SIGNALING_PATHWAY” were also generally recognized as cancer-related signaling pathway. These pathways were widely involved in tumor occurrence and development. The mechanism of these signaling pathways involved in *NOS3* regulation of gastric cancer needs further study.

Currently, research on *NOS3*-targeted medicine is mainly concentrating on cardiovascular and cerebrovascular disease. Many inhibitors and agonists have been found to have satisfactory therapeutic effects. For example, ursolic acid, which has an anti-tumor effect, has been proven to promote *NOS3* phosphorylation and inhibit *NOS3* uncoupling, thereby preventing doxorubicin-induced cardiac toxicity (50). However, the research on and application of *NOS3*-targeted medicine in malignant tumors are still extremely limited. The *NOS3* inhibitor L-NIO was reported to inhibit COAD cell growth and angiogenesis. Another *NOS3* inhibitor, N(G)-nitro-L-arginine methyl ester (L-NAME) was also reported to inhibit PAAD tumor growth (15). In addition, L-NIO could promote the anti-tumor effect of lenvatinib (36, 37). The *NOS3* level was significantly correlated with outcomes of bevacizumab-based chemotherapy in COAD (9, 51). Unfortunately, bevacizumab, L-NIO, and L-NAME were not included in the CTRP database. Our research showed that “SR8278,” an antagonist of Rev-ErbAα was negatively correlated with *NOS3* expression, indicating that *NOS3* was the potential target of “SR8278.” “SR8278” targeted NR1D1, a nuclear hormone receptor (52), reiterating the potential relationship between *NOS3* and NR1D1. Further analyses in STAD showed that response of STAD cancer cells to QS-11 and brivanib were strongly correlated with *NOS3* expression. QS-11 is an inhibitor of GTPase activating protein of ARF (ARFGAP), increasing ARF1-GTP and ARF6-GTP levels (53). Currently, studies on QS-11 in malignant tumor are very few. Only one research by Zhang et al. reported that the combination of QS-11 and ARFGAP1 protein could stimulate Wnt/β-catenin signaling pathway, resulting in the regulation of cell differentiation, proliferation, and apoptosis (54). Our research suggested that QS-11 may play an important role in STAD by inhibiting NOS3, which requires further research in the future. Brivanib is a dual tyrosine kinase inhibitor used to treat solid tumor in advanced stages. It can selectively target vascular endothelial growth factor receptor (VEGFR) and fibroblast growth factor receptor (FGFR) (55, 56). However, the response of *NOS3* expressed cell to brivanib has not been reported. *NOS3* was a downstream molecular of VEGFR signaling pathway. Thus, we guessed that brivanib might regulate *NOS3* through VEGFR signaling pathway. These results about drug response warrant further investigation.

In conclusion, this research showed that the expression level and clinical significance of *NOS3* was highly cancer-dependent. Analyses in public data sets gastric cancer tissues demonstrated that higher *NOS3* expression was related to poor prognosis of patients with STAD. *NOS3* was an independent prognostic factor for patients with STAD. Increased expression of *NOS3* might influence occurrence and development of STAD through several canonical cancer-related pathways. In addition, *NOS3* expression was related to some therapeutic drugs, such as “SR8278” and “brivanib,” which warrant further investigation. These results reported that *NOS3* might participate in occurrence and development of gastric cancer by canonical signaling pathways, suggesting that NOS3 might a novel target for gastric cancer treatment.

## Data Availability Statement

Publicly available datasets were analyzed in this study. This data can be found at: UCSC Xena (https://xena.ucsc.edu/), CCLE (CCLE; https://portals.broadinstitute.org/ccle/), and CTRP (https://portals.broadinstitute.org/ctrp.v2.1/).

## Ethics Statement

The studies involving human participants were reviewed and approved by the ethics committee of the First Affiliated Hospital of China Medical University. The patients/participants provided their written informed consent to participate in this study.

## Author Contributions

YZ, YT, and ZJ: project administration. ZL, FL, and JZ: software. DZ, YC, YM, and ZJ: statistical analysis. JS, CY, and YJ: visualization. DZ and YY: manuscript writing. All authors: contributed to the article and approved the submitted version.

## Conflict of Interest

The authors declare that the research was conducted in the absence of any commercial or financial relationships that could be construed as a potential conflict of interest.

## Supplementary Material

Supplementary Figure 1 NOS3 mRNA expression level in normal tissues from GTEx database.

Supplementary Figure 2 A scatter plot of *NOS3* mRNA and *TNF-*α mRNA. The correlation between two variables is analyzed by Spearman analysis.

Supplementary Figure 3 Association between *NOS3* mRNA expression and tumor stage in STAD. ANOVA analysis showed that tumor stages (stage I-IV) were not related to NOS3 expression, with *p*-value more than 0.05 (mean ± SD: I, 9.63 ± 1.139; II, 10.01 ± 0.8962; III, 9.845 ± 1.014; IV,9.983 ± 1.075) **(A)**. *T*-test also suggested that tumor stages (stage I-II and stage III-IV) showed no connection with NOS3 expression, with *p*-value more than 0.05 (mean ± SD: I-II, 9.888 ± 0.9934; III-IV, 9.872 ± 1.025) **(B)**.

Supplementary Figure 4 Association between *NOS3* mRNA expression and tumor stage in COAD **(A)** and BRCA **(B)** (^*^*p* < 0.05, ^**^*p* < 0.01).

Supplementary Table 1 Case number of all the tumor types.

## References

[1] Xu W Liu LZ Loizidou M Ahmed M Charles IG. The role of nitric oxide in cancer. Cell Res. (2002) 12:311–20. 10.1038/sj.cr.7290133 12528889

[2] Fischmann TO Hruza A Niu XD Fossetta JD Lunn CA Dolphin E. Structural characterization of nitric oxide synthase isoforms reveals striking active-site conservation. Nat Struct Biol. (1999) 6:233–42. 10.1038/6675 10074942

[3] Trane AE Pavlov D Sharma A Saqib U Lau K van Petegem F. Deciphering the binding of caveolin-1 to client protein endothelial nitric-oxide synthase (eNOS): scaffolding subdomain identification, interaction modeling, and biological significance. J Biol Chem. (2014) 289:13273–83. 10.1074/jbc.M113.528695 24648521 PMC4036337

[4] Pritchard KA Ackerman AW Ou J Curtis M Smalley DM Fontana JT. Native low-density lipoprotein induces endothelial nitric oxide synthase dysfunction: role of heat shock protein 90 and caveolin-1. Free Radical Biol Med. (2002) 33:52–62. 10.1016/S0891-5849(02)00851-1 12086682

[5] Xia N Daiber A Habermeier A Closs EI Thum T Spanier G. Resveratrol reverses endothelial nitric-oxide synthase uncoupling in apolipoprotein E knockout mice. J Pharmacol Exp Therap. (2010) 335:149–54. 10.1124/jpet.110.168724 20610621

[6] Ying L Hofseth LJ. An emerging role for endothelial nitric oxide synthase in chronic inflammation and cancer. Cancer Res. (2007) 67:1407–10. 10.1158/0008-5472.CAN-06-2149 17308075

[7] Song Y Zhao XP Song K Shang ZJ. Ephrin-A1 is up-regulated by hypoxia in cancer cells and promotes angiogenesis of HUVECs through a coordinated cross-talk with eNOS. PLoS ONE. (2013) 8:e74464. 10.1371/journal.pone.0074464 24040255 PMC3767678

[8] Zhang L Zeng M Fu BM. Inhibition of endothelial nitric oxide synthase decreases breast cancer cell MDA-MB-231 adhesion to intact microvessels under physiological flows. Am J Physiol Heart Circul Physiol. (2016) 310:H1735–47. 10.1152/ajpheart.00109.2016 27059076 PMC4935524

[9] Marisi G Scarpi E Passardi A Nanni O Ragazzini A Valgiusti M. Circulating VEGF and eNOS variations as predictors of outcome in metastatic colorectal cancer patients receiving bevacizumab. Sci Rep. (2017) 7:1293. 10.1038/s41598-017-01420-0 28465540 PMC5431064

[10] Penarando J Lopez-Sanchez LM Mena R Guil-Luna S Conde F Hernandez V. A role for endothelial nitric oxide synthase in intestinal stem cell proliferation and mesenchymal colorectal cancer. BMC Biol. (2018) 16:3. 10.1186/s12915-017-0472-5 29329541 PMC5795284

[11] Maniyar R Chakraborty S Suriano R. Ethanol enhances estrogen mediated angiogenesis in breast cancer. J Cancer. (2018) 9:3874–85. 10.7150/jca.25581 30410590 PMC6218769

[12] Sharma S Guru SK Manda S Kumar A Mintoo MJ Prasad VD. A marine sponge alkaloid derivative 4-chloro fascaplysin inhibits tumor growth and VEGF mediated angiogenesis by disrupting PI3K/Akt/mTOR signaling cascade. Chem Biol Interact. (2017) 275:47–60. 10.1016/j.cbi.2017.07.017 28756150

[13] Gajalakshmi P Priya MK Pradeep T Behera J Muthumani K Madhuwanti S. Breast cancer drugs dampen vascular functions by interfering with nitric oxide signaling in endothelium. Toxicol Appl Pharmacol. (2013) 269:121–31. 10.1016/j.taap.2013.03.011 23531514

[14] Lim KH Ancrile BB Kashatus DF Counter CM. Tumour maintenance is mediated by eNOS. Nature. (2008) 452:646–9. 10.1038/nature06778 18344980 PMC2688829

[15] Lampson BL Kendall SD Ancrile BB Morrison MM Shealy MJ Barrientos KS. Targeting eNOS in pancreatic cancer. Cancer Res. (2012) 72:4472–82. 10.1158/0008-5472.CAN-12-0057 22738914 PMC3749841

[16] Yu S Jia L Zhang Y Wu D Xu Z Ng CF. Increased expression of activated endothelial nitric oxide synthase contributes to antiandrogen resistance in prostate cancer cells by suppressing androgen receptor transactivation. Cancer Lett. (2013) 328:83–94. 10.1016/j.canlet.2012.09.006 22995070

[17] Nanni S Aiello A Re A Guffanti A Benvenuti V Colussi C. Estrogen-dependent dynamic profile of eNOS-DNA associations in prostate cancer. PLoS ONE. (2013) 8:e62522. 10.1371/journal.pone.0062522 23658738 PMC3643940

[18] Trachootham D Chen G Zhang W Lu W Zhang H Liu J. Loss of p53 in stromal fibroblasts promotes epithelial cell invasion through redox-mediated ICAM1 signal. Free Radical Biol Med. (2013) 58:1–13. 10.1016/j.freeradbiomed.2013.01.011 23376231 PMC3622735

[19] Villegas SN Gombos R Garcia-Lopez L Gutierrez-Perez I Garcia-Castillo J Vallejo DM. PI3K/Akt cooperates with oncogenic notch by inducing nitric oxide-dependent inflammation. Cell Rep. (2018) 22:2541–9. 10.1016/j.celrep.2018.02.049 29514083

[20] Li Q Wei X Zhou ZW Wang SN Jin H Chen KJ. GADD45alpha sensitizes cervical cancer cells to radiotherapy via increasing cytoplasmic APE1 level. Cell Death Dis. (2018) 9:524. 10.1038/s41419-018-0452-x 29743554 PMC5943293

[21] Su CW Chien MH Lin CW Chen MK Chow JM Chuang CY. Associations of genetic variations of the endothelial nitric oxide synthase gene and environmental carcinogens with oral cancer susceptibility and development. Nitric Oxide. (2018) 79:1–7. 10.1016/j.niox.2018.06.005 29932969

[22] Zhu Y Jiang H Chen Z Lu B Li J Peng Y. The genetic association between iNOS and eNOS polymorphisms and gastric cancer risk: a meta-analysis. Onco Targets Therap. (2018) 11:2497–507. 10.2147/OTT.S161925 29765229 PMC5939909

[23] Di Salvatore M Lo Giudice L Rossi E Santonocito C Nazzicone G Rodriquenz MG. Association of IL-8 and eNOS polymorphisms with clinical outcomes in bevacizumab-treated breast cancer patients: an exploratory analysis. Clin Trans Oncol. (2016) 18:40–6. 10.1007/s12094-015-1334-7 26141413

[24] Smeda M Kieronska A Adamski MG Proniewski B Sternak M Mohaissen T. Nitric oxide deficiency and endothelial-mesenchymal transition of pulmonary endothelium in the progression of 4T1 metastatic breast cancer in mice. Breast Cancer Res. (2018) 20:86. 10.1186/s13058-018-1013-z 30075800 PMC6091065

[25] Zheng Y Dai Y Liu W Wang N Cai Y Wang S. Astragaloside IV enhances taxol chemosensitivity of breast cancer via caveolin-1-targeting oxidant damage. J Cell Physiol. (2019) 234:4277–90. 10.1002/jcp.27196 30146689

[26] Vivian J Rao AA Nothaft FA Ketchum C Armstrong J Novak A. Toil enables reproducible, open source, big biomedical data analyses. Nat Biotechnol. (2017) 35:314–6. 10.1038/nbt.3772 28398314 PMC5546205

[27] Choi S Kim J Kim JH Lee DK Park W Park M. Carbon monoxide prevents TNF-α-induced eNOS downregulation by inhibiting NF-κB-responsive miR-155-5p biogenesis. Exp Mol Med. (2017) 49:e403. 10.1038/emm.2017.193 29170479 PMC5704195

[28] Ju Q Li X Zhang H Yan S Li Y Zhao Y. NFE2L2 is a potential prognostic biomarker and is correlated with immune infiltration in brain lower grade glioma: a pan-cancer analysis. Oxid Med Cell Longev. (2020) 2020:3580719. 10.1155/2020/3580719 33101586 PMC7569466

[29] ICGC/TCGA Pan-Cancer Analysis of Whole Genomes Consortium. Pan-cancer analysis of whole genomes. Nature. (2020) 578:82–93. 10.1038/s41586-020-1969-6 32025007 PMC7025898

[30] PCAWG Transcriptome Core Group Calabrese C Davidson NR Demircioglu D Fonseca NA He Y. Genomic basis for RNA alterations in cancer. Nature. (2020) 578:129–36. 10.1038/s41586-020-1970-0 32025019 PMC7054216

[31] Laufs U Werner N Link A Endres M Wassmann S Jurgens K. Physical training increases endothelial progenitor cells, inhibits neointima formation, and enhances angiogenesis. Circulation. (2004) 109:220–6. 10.1161/01.CIR.0000109141.48980.37 14691039

[32] Gertz K Priller J Kronenberg G Fink KB Winter B Schrock H. Physical activity improves long-term stroke outcome via endothelial nitric oxide synthase-dependent augmentation of neovascularization and cerebral blood flow. Circul Res. (2006) 99:1132–40. 10.1161/01.RES.0000250175.14861.77 17038638

[33] Krause BJ Peñaloza E Candia A Cañas D Hernández C Arenas GA. Adult vascular dysfunction in foetal growth-restricted guinea-pigs is associated with a neonate-adult switching in Nos3 DNA methylation. Acta Physiol. (2019) 227:e13328. 10.1111/apha.13328 31177629

[34] Zarrei M MacDonald JR Merico D Scherer SW. A copy number variation map of the human genome. Nat Rev Genet. (2015) 16:172–83. 10.1038/nrg3871 25645873

[35] Panich U Tangsupa-a-nan V Onkoksoong T Kongtaphan K Kasetsinsombat K Akarasereenont P. Inhibition of UVA-mediated melanogenesis by ascorbic acid through modulation of antioxidant defense and nitric oxide system. Arch Pharm Res. (2011) 34:811–20. 10.1007/s12272-011-0515-3 21656367

[36] Altun A Temiz TK Balci E Polat ZA Turan M. Effects of tyrosine kinase inhibitor E7080 and eNOS inhibitor L-NIO on colorectal cancer alone and in combination. Chinese J Cancer Res. (2013) 25:572–84. 10.3978/j.issn.1000-9604.2013.10.10 24255582 PMC3828440

[37] Gao Y Zhou S Xu Y Sheng S Qian SY Huo X. Nitric oxide synthase inhibitors 1400W and L-NIO inhibit angiogenesis pathway of colorectal cancer. Nitric Oxide. (2019) 83:33–9. 10.1016/j.niox.2018.12.008 30590117 PMC6677402

[38] Doi C Noguchi Y Marat D Saito A Fukuzawa K Yoshikawa T. Expression of nitric oxide synthase in gastric cancer. Cancer Lett. (1999) 144:161–7. 10.1016/S0304-3835(99)00222-0 10529016

[39] Wang L Shi GG Yao JC Gong W Wei D Wu TT. Expression of endothelial nitric oxide synthase correlates with the angiogenic phenotype of and predicts poor prognosis in human gastric cancer. Gastric Cancer. (2005) 8:18–28. 10.1007/s10120-004-0310-7 15747170

[40] Robey RW Pluchino KM Hall MD Fojo AT Bates SE Gottesman MM. Revisiting the role of ABC transporters in multidrug-resistant cancer. Nat Rev Cancer. (2018) 18:452–64. 10.1038/s41568-018-0005-8 29643473 PMC6622180

[41] Fletcher JI Williams RT Henderson MJ Norris MD Haber M. ABC transporters as mediators of drug resistance and contributors to cancer cell biology. Drug Resist Updates. (2016) 26:1–9. 10.1016/j.drup.2016.03.001 27180306

[42] Monteith GR Prevarskaya N Roberts-Thomson SJ. The calcium-cancer signalling nexus. Nat Rev Cancer. (2017) 17:367–80. 10.1038/nrc.2017.18 28386091

[43] Berridge MJ. The inositol trisphosphate/calcium signaling pathway in health and disease. Physiol Rev. (2016) 96:1261–96. 10.1152/physrev.00006.2016 27512009

[44] Huang Q Cao H Zhan L Sun X Wang G Li J. Mitochondrial fission forms a positive feedback loop with cytosolic calcium signaling pathway to promote autophagy in hepatocellular carcinoma cells. Cancer Lett. (2017) 403:108–18. 10.1016/j.canlet.2017.05.034 28624623

[45] Gregório C Soares-Lima SC Alemar B Recamonde-Mendoza M Camuzi D de Souza-Santos PT. Calcium signaling alterations caused by epigenetic mechanisms in pancreatic cancer: from early markers to prognostic impact. Cancers. (2020) 12:1735. 10.3390/cancers12071735 32629766 PMC7407273

[46] Jin SW Choi CY Hwang YP Kim HG Kim SJ Chung YC. Betulinic acid increases eNOS phosphorylation and NO synthesis via the calcium-signaling pathway. J Agric Food Chem. (2016) 64:785–91. 10.1021/acs.jafc.5b05416 26750873

[47] Najafi M Farhood B Mortezaee K. Extracellular matrix (ECM) stiffness and degradation as cancer drivers. J Cell Biochem. (2019) 120:2782–90. 10.1002/jcb.27681 30321449

[48] Marsico G Russo L Quondamatteo F Pandit A. glycosylation and integrin regulation in cancer. Trends Cancer. (2018) 4:537–52. 10.1016/j.trecan.2018.05.009 30064662

[49] Njah K Chakraborty S Qiu B Arumugam S Raju A Pobbati AV. A role of agrin in maintaining the stability of vascular endothelial growth factor receptor-2 during tumor angiogenesis. Cell Rep. (2019) 28:949–65.e7. 10.1016/j.celrep.2019.06.036 31340156

[50] Mu H Liu H Zhang J Huang J Zhu C Lu Y. Ursolic acid prevents doxorubicin-induced cardiac toxicity in mice through eNOS activation and inhibition of eNOS uncoupling. J Cell Mol Med. (2019) 23:2174–83. 10.1111/jcmm.14130 30609217 PMC6378202

[51] Ulivi P Scarpi E Passardi A Marisi G Calistri D Zoli W. eNOS polymorphisms as predictors of efficacy of bevacizumab-based chemotherapy in metastatic colorectal cancer: data from a randomized clinical trial. J Trans Med. (2015) 13:258. 10.1186/s12967-015-0619-5 26259598 PMC4531503

[52] Ferder IC Fung L Ohguchi Y Zhang X Lassen KG Capen D. Meiotic gatekeeper STRA8 suppresses autophagy by repressing Nr1d1 expression during spermatogenesis in mice. PLoS Genet. (2019) 15:e1008084. 10.1371/journal.pgen.1008084 31059511 PMC6502318

[53] Kanamarlapudi V Thompson A Kelly E López Bernal A. ARF6 activated by the LHCG receptor through the cytohesin family of guanine nucleotide exchange factors mediates the receptor internalization and signaling. J Biol Chem. (2012) 287:20443–55. 10.1074/jbc.M112.362087 22523074 PMC3370224

[54] Singh MK Gao H Sun W Song Z Schmalzigaug R Premont RT. Structure-activity relationship studies of QS11, a small molecule Wnt synergistic agonist. Bioorganic Med Chem Lett. (2015) 25:4838–42. 10.1016/j.bmcl.2015.06.062 26152429 PMC4607626

[55] Hofman J Sorf A Vagiannis D Sucha S Kammerer S Küpper JH. Brivanib Exhibits Potential for Pharmacokinetic Drug-Drug Interactions and the Modulation of Multidrug Resistance through the Inhibition of Human ABCG2 Drug Efflux Transporter and CYP450 Biotransformation Enzymes. Molecular pharmaceutics. (2019) 16:4436–50. 10.1021/acs.molpharmaceut.9b00361 31633365 PMC12379003

[56] Diaz-Padilla I Siu LL. Brivanib alaninate for cancer. Expert Opinion Investigat Drugs. (2011) 20:577–86. 10.1517/13543784.2011.565329 21391890

[57] Zou D Lv F Yang Y Yang C Chen Y Jin Z. Abnormal expression of NOS3 and association with clinical outcome are highly cancer dependent as revealed through pan-cancer analysis. Research Square [Preprint]. (2019) 1–20. 10.21203/rs.2.19006/v1

