# Supplementary figures and images for: Pan-Cancer Analysis of NOS3 Identifies Its Expression and Clinical Relevance in Gastric Cancer
Source: Front Oncol. 2021 Mar 4;11:592761. doi: 10.3389/fonc.2021.592761 (PMC7969995; doi:10.3389/fonc.2021.592761)

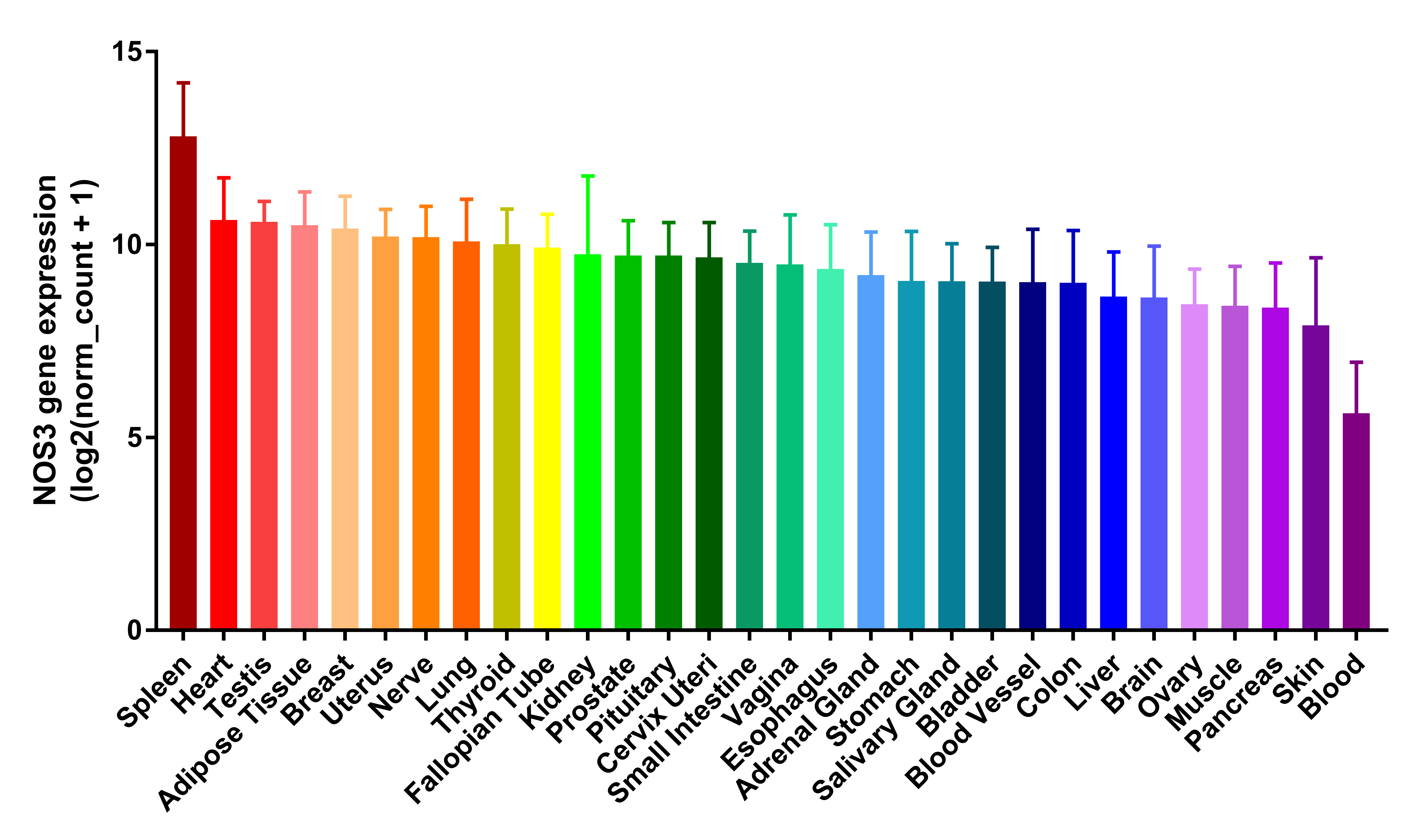

Supplement: Supplementary Figure 1 — NOS3 mRNA expression level in normal tissues from GTEx database. [file Image_1.TIF]

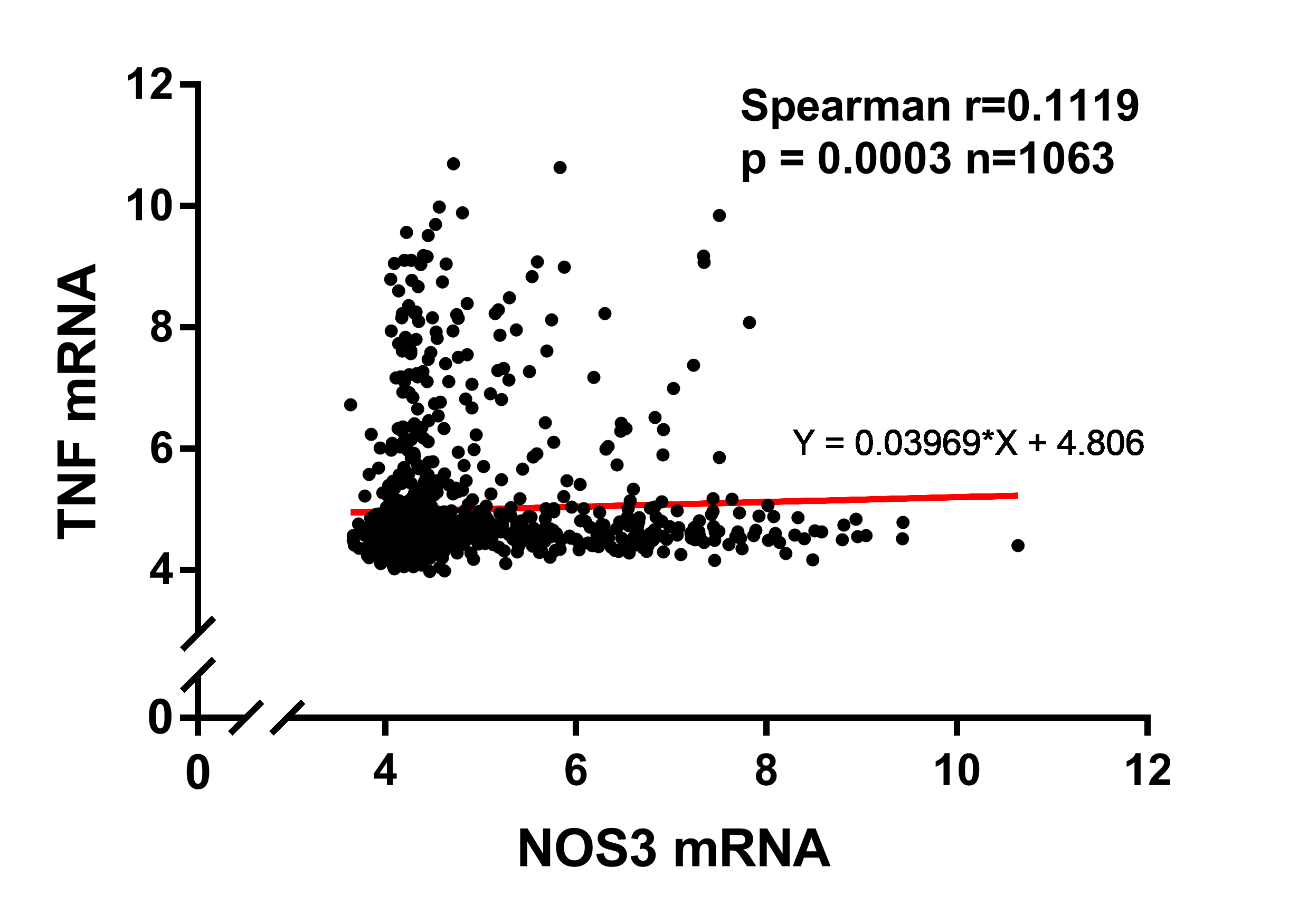

Supplement: Supplementary Figure 2 — A scatter plot of NOS3 mRNA and TNF-α mRNA. The correlation between two variables is analyzed by Spearman analysis. [file Image_2.TIF]

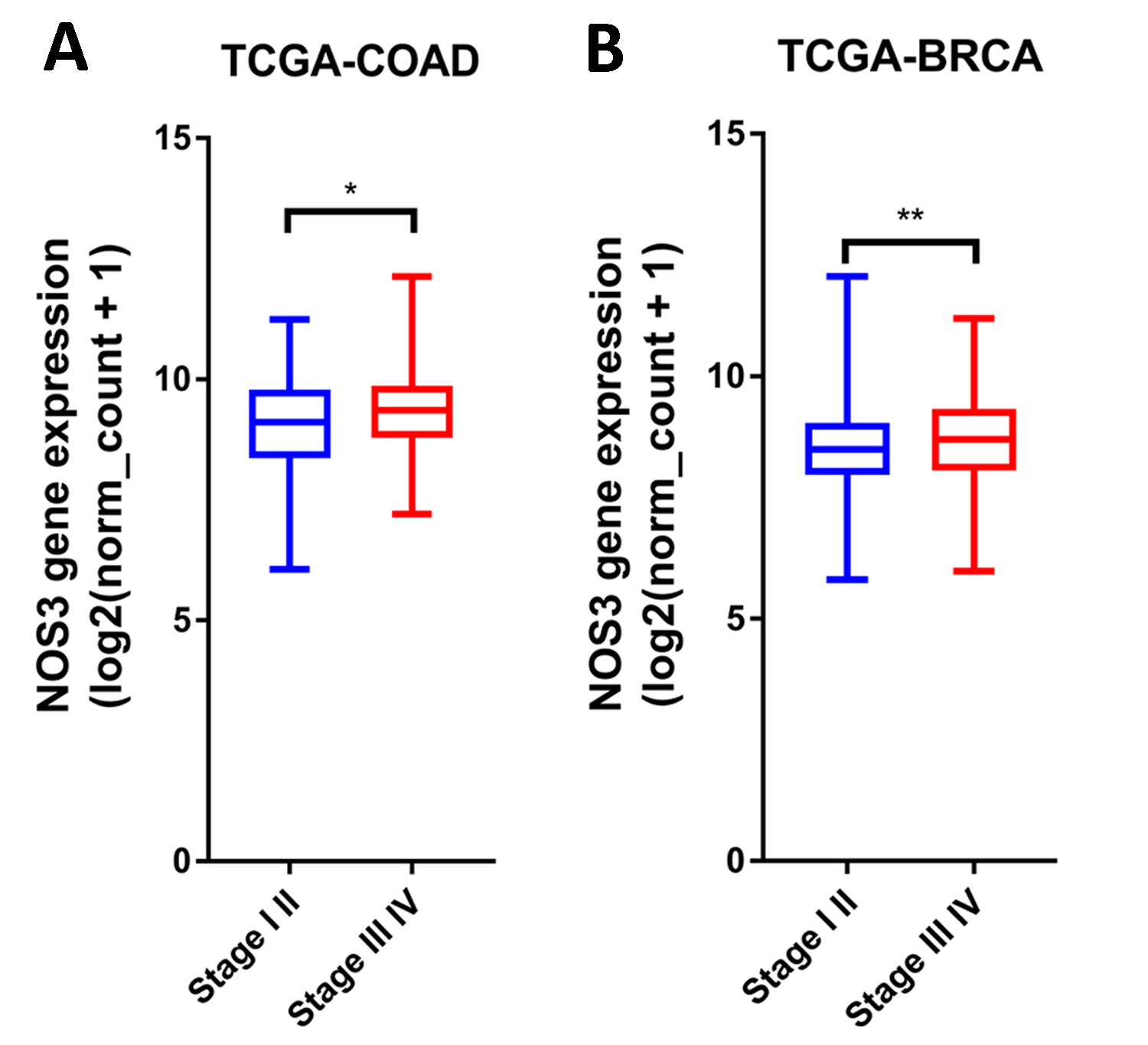

Supplement: Supplementary Figure 4 — Association between NOS3 mRNA expression and tumor stage in COAD (A) and BRCA (B) (*p < 0.05, **p < 0.01). [file Image_4.TIF]
