# Supplementary material for: Pan-Cancer Analysis of NOS3 Identifies Its Expression and Clinical Relevance in Gastric Cancer
Source: Front Oncol. 2021 Mar 4;11:592761. doi: 10.3389/fonc.2021.592761 (PMC7969995; doi:10.3389/fonc.2021.592761)
Supplement: Supplementary Table 1 — Case number of all the tumor types. [file Table_1.DOCX]

| **Disease Name** | **Cohort** | **Tumor** | **Normal** |
| --- | --- | --- | --- |
| Adrenocortical carcinoma | ACC | 77 | / |
| Bladder urothelial carcinoma | BLCA | 407 | 19 |
| Breast invasive carcinoma | BRCA | 1092 | 113 |
| Cervical and endocervical cancers | CESC | 304 | 3 |
| Cholangiocarcinoma | CHOL | 36 | 9 |
| Colon adenocarcinoma | COAD | 288 | 41 |
| Lymphoid Neoplasm Diffuse Large B-cell Lymphoma | DLBC | 48 | / |
| Esophageal carcinoma | ESCA | 181 | 13 |
| Glioblastoma multiforme | GBM | 166 | 5 |
| Head and Neck squamous cell carcinoma | HNSC | 518 | 444 |
| Kidney Chromophobe | KICH | 66 | 25 |
| Kidney renal clear cell carcinoma | KIRC | 530 | 72 |
| Kidney renal papillary cell carcinoma | KIRP | 288 | 32 |
| Acute Myeloid Leukemia | LAML | 173 | / |
| Brain Lower Grade Glioma | LGG | 509 | / |
| Liver hepatocellular carcinoma | LIHC | 369 | 50 |
| Lung adenocarcinoma | LUAD | 513 | 59 |
| Lung squamous cell carcinoma | LUSC | 498 | 50 |
| Mesothelioma | MESO | 87 | / |
| Ovarian serous cystadenocarcinoma | OV | 420 | / |
| Pancreatic adenocarcinoma | PAAD | 178 | 4 |
| Pheochromocytoma and Paraganglioma | PCPG | 177 | 3 |
| Prostate adenocarcinoma | PRAD | 495 | 52 |
| Rectum adenocarcinoma | READ | 92 | 10 |
| Sarcoma | SARC | 258 | / |
| Skin Cutaneous Melanoma | SKCM | 102 | / |
| Stomach adenocarcinoma | STAD | 414 | 36 |
| Testicular Germ Cell Tumors | TGCT | 148 | / |
| Thyroid carcinoma | THCA | 503 | 59 |
| Thymoma | THYM | 119 | / |
| Uterine Corpus Endometrial Carcinoma | UCEC | 181 | 23 |
| Uterine Carcinosarcoma | UCS | 57 | / |
| Uveal Melanoma | UVM | 79 | / |
